# A needs-based method for estimating the behavioral health staff needs of community health centers

**DOI:** 10.1186/1472-6963-13-245

**Published:** 2013-07-02

**Authors:** Bridget Teevan Burke, Benjamin F Miller, Michelle Proser, Stephen M Petterson, Andrew W Bazemore, Eric Goplerud, Robert L Phillips

**Affiliations:** 1 Johns Hopkins Bloomberg School of Public Health, 615 N Wolfe St, Baltimore, MD 21205, USA; 2 Department of Family Medicine, University of Colorado Denver School of Medicine, Mail Stop F496 Academic Office 1, 12631 East 17th Avenue, Room 3403, Aurora, CO 80045, USA; 3 National Association of Community Health Centers, 1400 I Street, NW, Suite 910, Washington, DC 20005, USA; 4 The Robert Graham Center, 1133 Connecticut Ave NW, Suite 1100, Washington, DC 20036, USA; 5 Substance Abuse, Mental Health and Criminal Justice Studies, NORC at the University of Chicago, 4350 East West Highway 8th Floor, Bethesda, MD 20814, USA; 6 The American Board of Family Medicine, 1648 McGrathiana Parkway Suite 550, Lexington, KY 40511-1247, USA

## Abstract

**Background:**

Federally Qualified Health Centers are expanding to increase access for millions of more Americans with a goal of doubling capacity to serve 40 million people. Health centers provide a lot of behavioral health services but many have difficulty accessing mental health and substance use professionals for their patients. To meet the needs of the underserved and newly insured it is important to better estimate how many behavioral health professionals are needed.

**Methods:**

Using health center staffing data and behavioral health service patterns from the 2010 Uniform Data System and the 2010 National Survey on Drug Use and Health, we estimated the number of patients likely to need behavioral health care by insurance type, the number of visits likely needed by health center patients annually, and the number of full time equivalent providers needed to serve them.

**Results:**

More than 2.5 million patients, 12 or older, with mild or moderate mental illness, and more than 357,000 with substance abuse disorders, may have gone without needed behavioral health services in 2010. This level of need would have required more than 11,600 full time providers. This translates to approximately 0.9 licensed mental health provider FTE, 0.1 FTE psychiatrist, 0.4 FTE other mental health staff, and 0.3 FTE substance abuse provider per 2,500 patients. These estimates suggest that 90% of current centers could not access mental health services or provide substance abuse services to fully meet patients’ needs in 2010. If needs are similar after health center expansion, more than 27,000 full time behavioral health providers will be needed to serve 40 million medical patients, and grantees will need to increase behavioral health staff more than four-fold.

**Conclusions:**

More behavioral health is seen in primary care than in any other setting, and health center clients have greater behavioral health needs than typical primary care patients. Most health centers needed additional behavioral health services in 2010, and this need will be magnified to serve 40 million patients. Further testing of these workforce models are needed, but the degree of current underservice suggests that we cannot wait to move on closing the gap.

## Background

Federally-Qualified Health Centers (FQHCs) are non-profit, community-directed health centers that provide comprehensive health care to patients without regard to income or insurance coverage. Often referred to as Community Health Centers or simply “health centers”, these safety net providers offer primary and preventive care, mental health, substance use, dental, and pharmacy services to patients in high-need communities. Community health centers address the physical, mental and social needs of their patients by providing access to comprehensive health and social services.

Health centers eliminate many barriers to health care, including cost, lack of insurance, geographic location, language, and cultural competency for America’s most medically underserved populations. Health centers care for 20% of all low-income, uninsured Americans, and 1 in 7 Medicaid beneficiaries [1]. Approximately 93% of patients are at or below 200 percent of the federal poverty level (FPL) and 76% are either covered by Medicaid/CHIP or are uninsured [2]. Roughly two-thirds of patients are racial or ethnic minorities, and almost one-quarter of patients are best served in a language other than English [2].

Ensuring access to behavioral health services is a particularly important role for health centers since health center patients likely have rates of mental illness and substance use that are greater than the general population. Health centers serve a disproportionate number of uninsured, Medicaid insured, and patients below the federal poverty level compared to other primary care delivery sites. According to the National Survey on Drug Use and Health (NSDUH), these patients are more likely than the general population to have a behavioral health disorder. In 2010, nearly 30 percent of uninsured persons and those with incomes less than 100 percent of the federal poverty level had any mental illness in the past year, and almost one-third of Medicaid and CHIP enrollees over age 12 suffered from a mental illness [3]. Nearly 7 percent of uninsured respondents abused illicit drugs or alcohol in the past year [3]. In contrast, 20 percent of the general population had any past year mental illness in 2010, and 4.5% had a substance abuse disorder [3].

Given the unique potential of health centers to provide behavioral health services for the neediest populations, it is important to develop a method for quantifying how many behavioral health professionals are needed to adequately serve patients. With no national standard for behavioral health staffing, health centers vary significantly in the type and number of behavioral health professionals they employ. The Affordable Care Act provided $9.5 billion in funding over 5 years through a dedicated Health Center Fund, beginning in 2011, to allow health centers to expand their operational capacity with a goal of ensuring the newly insured would have access points for care. These expansion efforts, which have already commenced, provide an opportunity to plan for the staff that will treat the whole person. This paper presents a needs-based method for estimating the behavioral health staff needs of community health centers currently, and projects the need for additional providers as health centers expand to 40 million patients as a future target.

The numbers of health centers and patients served have grown significantly in the last two decades; there are currently over 1,200 health center organizations providing care to more than 20 million patients in over 8,100 locations dispersed across the nation [2,4,5]. Under full funding provided by the Affordable Care Act (ACA) of 2010 on top of health centers’ federal discretionary base and mandated Medicaid expansions in every state, health centers would have reached 40 million patients by 2015 [6]. Budget cuts to health centers’ baseline federal funding during FY 2011 significantly curtailed expansion. The Supreme Court’s ruling effectively changing the ACA’s Medicaid expansion into a state option rather than a mandate will also restrain health center growth in states that opt out. However, continued federal and state investments will allow the Health Center Program to grow.

### Community health centers and behavioral health

At a minimum, all health centers that receive grant funding from the Health Resources and Services Administration (HRSA) under Section 330 of the Public Health Service Act are required to provide referrals to substance abuse and mental health providers. Evidence suggests many grantees exceed this standard. Nearly three-quarters of health centers had onsite mental health or substance abuse staff in 2010, and they provided more than 5.2 million encounters [2]. Among encounters with a reported primary diagnosis in 2010, encounters for depression were the third most frequent, surpassed only by hypertension and diabetes [2]. Given that most patients are uninsured or Medicaid-insured, many of these patients likely would have gone unserved. A 2004 survey of health center directors revealed that health centers without onsite mental health services reported more difficulty accessing specialty mental health services compared to those that had these services onsite, particularly for their uninsured and Medicaid patients [7].

Although epidemiologic evidence suggests a substantial need for behavioral health services among low income, uninsured, and Medicaid-insured individuals, there are often significant barriers to receiving adequate treatment. Historically, the behavioral health sector has been segregated from the medical sector in organization and financing, and research suggests this fragmentation creates barriers to identifying and treating mental health problems, especially for uninsured and Medicaid insured patients [8,9] Half of all mental health problems in the U.S. population go undiagnosed, and a significant portion of patients diagnosed with a mental health or drug use disorder never receive treatment [10,11]. Hispanics, non-Hispanic blacks, and those with less education are less likely to obtain specialty mental health care than the general population [12-14]. Even when patients recognize a need for behavioral health services, many are unaware of their options for receiving that care, or have problems accessing the system due to availability of providers or financial barriers [15-18].

Under the current system, patients who seek behavioral health care overwhelmingly seek it from general medical providers, which have been described as the “de facto” mental health system in the U.S. [11,13,19]. Decades of research have demonstrated the inseparability of mental and physical health, and new models that bring mental health providers into the primary care setting as members of the health care team are proving effective at increasing the accessibility and acceptability of mental health treatment [20]. In fully integrated care, patients become accustomed to behavioral health care as a “routine part” of primary care [21]. Care provided in this manner has been shown to reduce stigma for patients, increase patient engagement, and reduce attrition after care is initiated [22-24]. Rural areas and underserved urban areas also benefit significantly from integrated care, since shortages of behavioral health providers create long wait times for patients needing appointments [16].

Despite a significant number of integrated behavioral health-primary care initiatives in the U.S., no standards exist for staffing. In 2003, Faulkner published a needs-based approach to modeling the psychiatric workforce based on disease prevalence, need for treatment and available resources [25]. In recent years, the federal government has recognized the need for behavioral health workforce planning at the national level to improve access and quality across distributed delivery sites. For example, five years after deploying the largest integrated behavioral health-primary care system in history, the Veterans Administration (VA) concluded delivery sites need more “central guidance on best practices in determining needed mental health staff” [26]. In 2007, the Department of Defense (DOD) Task Force on Mental Health recommended the DOD adopt a risk-adjusted population-based model for estimating the number and clinical specialty ratios for behavioral health providers in military medical departments [27]. The model, the Psychological Health Risk-Adjusted Model for Staffing, analyzes input on need for services among Defense Health Plan beneficiaries, provider productivity, and non-encounter based factors to project the number and ratio of mental health staff needed [28].

## Methods

**Figure 1:**
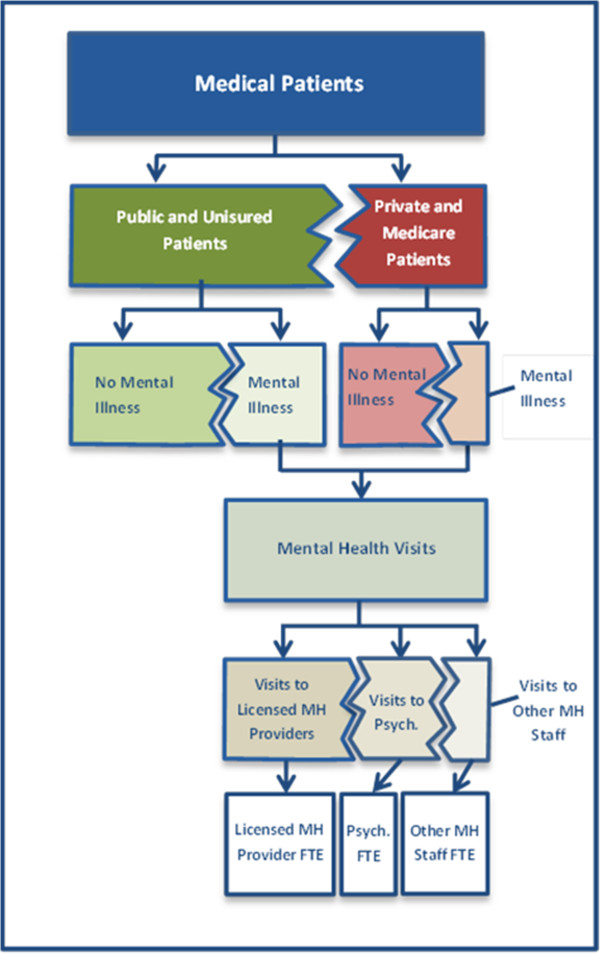
**Flow diagram for estimating FTE needed to serve mental health patients.** *Psych. refers to psychiatry.

We developed a method to estimate how many full time equivalent (FTE) behavioral health providers were needed across the health center program using data on prevalence of behavioral health disorders and existing staffing and utilization patterns among grantees. Using data on community health center staffing and service patterns from the 2010 Uniform Data System (UDS) and nationally representative data on mental illness and substance abuse from the 2010 National Survey on Drug Use and Health, we estimated the number of patients likely to need behavioral health care by insurance type, the number of visits likely needed by these patients in a year, and the number of full time equivalent providers needed to serve them (Figure 1).

The Uniform Data System is an annual collection of data by HRSA on health center organizational characteristics and performance. Recipients of grants under Section 330 of the Public Health Service Act are required to report UDS data annually. This study used counts of FTE psychiatrists, psychologists, licensed clinical social workers, other licensed mental health professionals (psychiatric social workers, psychiatric nurse practitioners, family therapists, and other licensed master’s degree-prepared clinicians), mental health staff (certified individuals who provide counseling, treatment or support services related to mental health professionals), and substance abuse staff from the UDS. The UDS also provided counts of medical patients, mental health patients and encounters by provider type, and the number of substance abuse patients and encounters.

Estimates of need for behavioral health services were derived from the 2010 National Survey on Drug Use and Health. Sponsored by the Substance Abuse and Mental Health Services Administration (SAMHSA), the NSDUH is an annual survey of approximately 70,000 individuals aged 12 or older across the U.S. on the use of illicit drugs, alcohol, tobacco, and mental health conditions. For this study, the NSDUH provided estimates of past year mental illness and illicit drug or alcohol abuse by insurance type, which included Medicare, Medicaid/CHIP, private insurance, and uninsured. The NSDUH creates a categorical mental illness indicator from responses to survey questions, including total scores from the psychological distress and impairment scales, the predicted probability of serious mental illness (SMI), and indicators of various levels of mental illness. Categories include no mental illness, mild mental illness, moderate mental illness, and serious mental illness. The NSDUH creates an indicator for illegal drug or alcohol abuse for respondents that provide a positive response to one or more criteria for alcohol, marijuana, cocaine, heroin, hallucinogen, inhalant, pain reliever, tranquilizer, stimulant, or sedative abuse. Respondents are either classified as positive or negative for substance abuse.

We started our estimation by examining annual behavioral health service utilization at the grantee level from the 2010 UDS (Table 1). The annual number of mental health visits per mental health patient and substance abuse visits per substance abuse patient were calculated for each grantee with onsite behavioral health staff. The distribution of mental health visits to psychiatrists, licensed mental health providers (psychologists, licensed clinical social workers, psychiatric social workers, psychiatric nurse practitioners, family therapists, and other licensed master’s degree-prepared clinicians), and other mental health staff was calculated by summing visits across grantees with onsite mental health staff and calculating the percent of visits to each provider type. We estimated the annual visit load for each provider type by dividing the number of visits in each provider category by the number of FTE for grantees with onsite behavioral health staff.

**Table 1:** Annual behavioral health service utilization, 2010

Median visits per mental health patient	3.7
Percent of visits to licensed MH providers^*^	59.9%
Percent of visits to psychiatrists	23.3%
Percent of visits to other mental health staff^**^	16.8%
Median visits per licensed MH provider^**^ FTE	889
Median visits per psychiatrist FTE	2,210
Median visits per other mental health staff FTE	648
Median visits per substance abuse*** patient	5.8
Median visits per substance abuse*** provider FTE	1,012

*Psychologists, licensed clinical social workers, and other licensed mental health professionals were grouped together for analyses as “licensed mental health providers”.

**Includes unlicensed individuals, including “certified” individuals who provide counseling, treatment or support services related to mental health professionals.

The UDS reports the number of patients by medical insurance type for two age categories, ages 0-19 and ages 20+. Across all health centers in 2010, 35.3% of patients were ages 0-19, and 64.7% were age 20 or older. Of patients age 0-19, 64.5% were age 0-11, and 35.5% were age 12-19. This age distribution was used to calculate the number of patients by insurance type ages 0-11 and ages 12-19 for each grantee. We selected only those age 12 and over for our analysis, since the NSDUH provides mental health and substance abuse prevalence data on persons age 12 and over.

***The UDS defines a substance abuse visit as “a visit between a substance abuse provider (e.g., a mental health provider or a credentialed substance abuse counselor, rehabilitation therapist, psychologist) and a patient during which alcohol or drug abuse services (i.e., assessment and diagnosis, treatment, or after care) are provided”. The UDS defines substance abuse services as “psychiatric nurses, psychiatric social workers, mental health nurses, clinical psychologists, clinical social workers, and family therapists and other individuals providing counseling and/or treatment services related to substance abuse”.

### Estimating the need for mental health staff

Next, we estimated the need for mental health services among medical patients age 12 and over. We selected health center medical patients as opposed to all health center patients (e.g. dental patients) because we assume most mental health and substance abuse patients are also medical patients, and more importantly, because health centers aim to integrate these services within physical health care to address patients’ full and inter-related health care needs.

**Figure 2:**
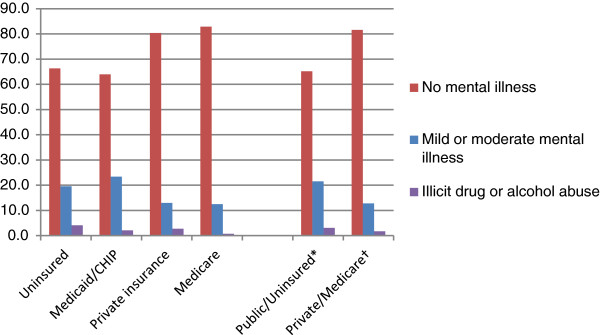
**Past year prevalence of mild or moderate mental illness or illicit drug or alcohol abuse by insurance type.** * Average past year mental illness and illicit drug or alcohol abuse for public insurance and uninsured. † Average past year mental illness and illicit drug or alcohol abuse for private insurance and Medicare. (Source: 2010 National Survey on Drug Use and Health). ^#^ Psychologists, licensed clinical social workers, and other licensed mental health professionals were grouped together for analyses as “licensed mental health providers”.

The NSDUH indicates those covered by Medicare have similar rates of mental illness to those with private insurance. Similarly, the uninsured and those with Medicaid/CHIP have similar rates of mental illness (Figure 2). We used insurance data from the UDS to group medical patients for each grantee into two categories: 1) Medicaid/CHIP/Other public insurance and uninsured, and 2) Medicare and private insurance. Applying prevalence data of past year mental illness by insurance type from the NSDUH, we estimated the number of patients age 12 and over likely to have mild or moderate mental illness , our target population for mental health treatment. Estimates were calculated for all grantees, including those without current mental health staff or patients. We excluded serious mental illness from this analysis because we assumed patients with SMI had a high likelihood of receiving mental health care in other safety net mental health settings, and the median number of visits per patient would greatly underestimate the need for providers to serve this population.

Using the median annual number of visits per mental health patient, we calculated the annual number of visits likely needed by patients with mild or moderate mental illness for all grantees. We divided these visits into those provided by licensed mental health providers, psychiatrists, and other mental health staff using the distribution of visits by provider type from the UDS. We divided the number of visits required by each provider type by the estimated annual visit load to determine the total number of FTE needed per grantee. Provider shortages were calculated by subtracting existing FTE from estimates of needed FTE.

### Estimating need for substance abuse staff

We estimated the need for substance abuse services by applying prevalence data of past year illicit drug or alcohol abuse by insurance type from the NSDUH to the two groups of medical patients, Medicaid/CHIP/Other public insurance and uninsured, and Medicare and private insurance (Figure 2). We excluded patients with illicit drug or alcohol dependence from this analysis. Patients with dependence are different from patients with abuse and likely receive treatment in inpatient specialty settings. Using the median annual number of visits per substance abuse patient from the UDS, we calculated the annual number of visits likely needed by patients age 12 and over at each grantee, including those currently without onsite staff or patients. We divided the number of visits required by the estimated annual visit load per substance abuse FTE to determine the total number of FTE needed per grantee. Provider shortages were calculated by subtracting existing FTE from estimates of needed FTE.

### Expansion to 40 million patients

We then used our method to project the need for mental health providers at health center capacity of 40 million medical patients. Given the Supreme Court’s ruling of the ACA, states now have the option of expanding Medicaid eligibility to all U.S. citizens with incomes up to 133 percent of the federal poverty level. Under the ACA, those with incomes up to 400 percent of the FPL will receive subsidies to purchase private insurance through the exchanges, starting in 2014. This will change the distribution of insurance at health centers. Models suggest the vast majority of new Medicaid enrollees will be previously uninsured, but estimates vary based on their assumptions [29]. Research also suggests income fluctuations will cause people to cycle between Medicaid and the insurance exchanges as their eligibility changes [30]. Although changes in the ACA and health center federal funding may restrain their growth, we maintain 40 million as a reasonable patient goal given substantial levels of unmet health care needs across the United States.

Given the uncertainty over how the ACA will alter the insurance coverage of health center patients, we made several assumptions for our projection. Since 72 percent of patients were below 100 percent of the FPL and 93% were below 200 percent of the FPL in 2010, we assumed a large portion of uninsured health center patients would become eligible for Medicaid. We also assumed the portion of patients covered by Medicare or private insurance would not change dramatically as the number of patients increased. Significantly fewer providers accept public insurance compared to those that accept private insurance or Medicare, giving Medicare and private insurance patients greater flexibility in choosing a provider. We assumed this would remain true under insurance expansion, and that health centers would continue to serve a large portion of patients on public insurance and a significant number of patients remaining without insurance.

### Behavioral health staff for 40 million patients

Using the 2010 UDS, we applied the mean percentage of patients on public insurance or uninsured by grantee to sort the 40 million medical patients into our two categories, Medicaid/CHIP/Other public insurance/uninsured, and Medicare/private insurance. Applying the average prevalence data of past year mild or moderate mental illness and illicit drug or alcohol abuse by insurance type from the NSDUH, we estimated the number of patients age 12 and over likely to need behavioral health treatment.

Using the median visit data from our model (Table 1), we estimated the annual number of visits likely needed by patients with mild or moderate mental illness, and substance abuse disorders. We divided mental health visits into those provided by licensed mental health providers, psychiatrists, and other mental health staff using the distribution of visits by provider type. We divided the number of visits required by each provider type by the annual visit load to determine the total number of FTE needed per grantee.

No IRB or ethical approval was sought for this study since it is a secondary analysis of using entirely public, de-identified data. We do not display U.S. territories and states with fewer than three grantees to avoid indirect identification.

## Results

The results from our model suggested a substantial number of medical patients age 12 and over likely had a need for behavioral health services in 2010 (Table 2). This included more than 2.5 million patients with mild or moderate mental illness, and more than 357,000 with illicit drug or alcohol abuse disorders. Using the median number of visits per behavioral health patient from the UDS, we estimated more than 11,600 behavioral health FTE were needed to serve them in 2010. The model suggested the need for approximately 0.9 licensed mental health provider FTE, 0.1 FTE psychiatrist, 0.4 FTE other mental health staff, and 0.3 FTE substance abuse provider per 2,500 medical patients.

**Table 2:** Estimated behavioral health staff needed to serve adults and adolescents in 2010

			**Shortage**	
	**Actual –2010 UDS**	Model results*	**Number of grantees**	Shortage**
Behavioral health patients				
Mental health	852,984	2,512,224	1,012	1,770,027
Substance abuse	98,760	357,632	1,002	302,613
Behavioral health FTE	5,094	11,699	-	8,777
Licensed mental health providers^#^	2,582	6,260	936	4,328
Psychiatrists	394	982	998	748
Other mental health staff	1,264	2,407	997	1,916
Substance abuse providers	854	2,050	998	1,785

*Estimated number based on the model used by the authors.

**Calculated after subtracting out patients and providers in the small number of grantees treating more patients than our model would suggest. This implies that some health centers have patients with particularly high needs for behavioral health services or many patients treated for behavioral health did not rely on the center for other medical care.

^#^ Psychologists, licensed clinical social workers, and other licensed mental health professionals were grouped together for analyses as “licensed mental health providers”.

According to this model, 1,012 grantees (90%) were unable to provide onsite mental health services to some segment of their patients in 2010 due to capacity constraints (Table 2). Across all grantees, an additional 1.7 million patients likely would have benefited from onsite mental health services, with an additional 6,992 mental health FTE (licensed MH providers, psychiatrists, and other MH staff) needed to serve them. The greatest absolute shortage was in licensed mental health provider FTE (4,328). The model suggested a need for as many as 2.5 times the number of psychiatrist FTE over existing staff across all grantees.

One hundred twelve grantees (10%) provided mental health care to a greater number of patients in 2010 than our method identified as a target for mental health treatment. These grantees served 110,000 more patients than the model identified as likely to have mild or moderate mental illness. This suggests that patients at these health centers had particularly high prevalence of mild or moderate mental illness, they treated patients with more severe mental illness, or patients were treated for mental health that did not use general medical services.

The model suggested 1,002 grantees (89%) needed to expand their onsite substance abuse services in order to fully meet patients’ needs in 2010 (Table 2). Across all grantees, an additional 302,000 patients likely would have benefited from onsite substance abuse services. An additional 1,785 substance abuse FTE would have been needed to serve them.

One hundred twenty-two (11%) grantees treated a greater number of patients for illicit drug or alcohol abuse in 2010 than our model identified as a target population for substance abuse treatment. These grantees served nearly 44,000 more patients than our model identified as likely to abuse illicit drugs or alcohol, again suggesting that some health center patient populations have higher than expected rates of illicit drug or alcohol abuse, grantees treated patients with more severe substance use problems, or patients were treated for substance use that did not use general medical services.

Assuming utilization patterns and provider productivity remain similar, we estimated more than 27,000 behavioral health FTE will be needed to serve 40 million medical patients (Table 3). The target number of behavioral health patients would be approximately 6.7 million annually. The model suggested grantees will need to increase behavioral health staff more than four-fold over 2010 levels to meet the needs of the additional patients.

**Table 3:** Estimates of need for behavioral health services and providers for 40 million medical patients

	**Target**	**Additional needed (% increase over 2010)**
Medical patients	40,000,000	23,222,848 (138%)
Mental health patients	5,918,248	5,065,264 (594%)
Substance abuse patients	841,182	742,422 (752%)
Behavioral health FTE	27,552	22,458 (441%)
Licensed mental health providers	14,748	12,166 (471%)
Psychiatrists	2,314	1,920 (487%)
Other mental health staff	5,669	4,405 (348%)
Substance abuse providers	4,821	3,967 (465%)

### State level findings

States and communities vary in their staffing needs given a wide variety of national, state, and local factors that influence provider location and practice decisions. Our analysis also generated state-level estimates of additional behavioral health staff currently needed in order to expand access to care for health center patients (Table 4).

**Table 4:** Estimates of the need for additional behavioral health patients and staff in 2010 by state^**1**^

**State**	**Mental health patients**	**Psychiatrist FTE**	**Licensed MH FTE**^**2**^	**Other MH FTE**^**3**^	**Substance abuse patients**	**Substance abuse providers**
AK	5,652	3.2	6.6	5.0	1,178	6.4
AL	39,916	16.0	100.6	38.2	5,116	30.8
AR	18,512	7.6	45.7	18.5	2,755	15.8
AZ	36,331	18.9	99.2	40.9	5,967	38.6
CA	298,138	118.8	779.9	280.6	46,093	277.3
CO	37,120	21.1	113.5	38.8	8,145	50.4
CT	17,656	4.0	20.0	18.0	3,797	21.9
DC	2,790	1.1	30.8	8.1	2,380	13.6
DE	3,498	1.7	8.2	4.3	535	3.1
FL	103,175	44.3	297.6	115.8	15,435	96.6
GA	41,670	16.0	98.7	40.9	5,668	30.6
HI	5,881	2.8	10.7	6.0	1,687	7.1
IA	17,687	7.6	42.2	18.7	2,730	15.8
ID	7,401	3.7	11.1	14.0	1,693	10.9
IL	104,439	39.1	283.8	117.5	20,697	115.4
IN	26,280	13.4	70.9	24.7	4,749	27.5
KS	9,814	4.7	17.5	12.3	1,833	9.4
KY	27,927	12.8	68.8	33.1	4,543	25.3
LA	13,704	7.0	30.1	20.0	3,219	16.7
MA	43,818	16.7	77.9	46.0	8,320	45.0
MD	22,866	7.8	40.0	27.6	3,289	17.4
ME	10,496	5.3	17.8	15.8	2,138	13.0
MI	47,030	23.8	100.1	58.8	8,409	46.7
MN	7,664	5.6	19.3	9.2	2,117	14.6
MO	27,773	11.8	60.1	29.1	4,611	31.1
MS	38,201	16.4	96.6	37.6	5,129	32.7
MT	6,054	4.5	13.5	8.0	1,199	6.9
NC	36,461	16.0	94.7	38.5	5,723	34.7
ND	3,019	1.3	7.0	3.1	358	2.2
NE	3,303	2.6	13.3	3.5	1,064	6.3
NH	6,405	3.4	13.5	7.0	703	4.5
NJ	49,345	20.2	120.0	47.4	7,565	44.3
NM	15,411	7.3	29.0	20.0	3,672	19.5
NY	111,123	36.4	216.4	133.5	18,775	114.7
OH	45,883	19.4	111.0	52.7	8,029	46.0
OK	12,080	5.1	25.7	13.6	2,220	13.9
OR	14,177	8.5	26.6	17.1	3,474	15.0
PA	50,328	17.2	126.2	42.8	8,405	58.4
PR	43,724	18.8	113.0	49.4	7,052	40.5
RI	9,254	5.3	20.5	11.5	2,054	11.8
SC	35,732	15.8	85.1	41.0	6,107	35.2
SD	6,747	2.8	16.4	7.2	1,055	6.0
TN	38,603	17.3	94.8	42.4	6,456	38.5
TX	95,741	36.4	237.4	112.5	16,446	99.4
UT	10,593	5.9	25.0	12.5	2,132	12.2
VA	27,912	12.7	59.1	32.1	4,713	26.7
VT	7,045	3.1	14.1	11.4	1,259	7.6
WA	55,700	29.3	140.6	47.6	9,566	55.7
WI	11,051	3.7	35.7	18.3	3,645	20.8
WV	36,414	15.6	86.2	42.1	5,675	32.0
WY	1,625	0.5	3.7	2.5	178	1.9

^1^States and U.S. territories with fewer than 3 grantees were excluded from this table.

^2^Psychologists, licensed clinical social workers, other licensed mental health professionals (psychiatric social workers, psychiatric nurse practitioners, family therapists, and other licensed master’s degree-prepared clinicians).

^3^Certified individuals who provide counseling, treatment or support services related to mental health professionals.

## Discussion

The results of this study suggested most health center grantees needed additional capacity for providing onsite behavioral health services in 2010, and the current constraints are likely to be magnified as health centers expand to 40 million patients. This may be reflective of constraints in the behavioral health workforce in the U.S. in general. Shortages of providers, particularly psychiatrists, have been described, and the workforce is aging as fewer graduates are entering some behavioral health professions [31,32]. The need for additional providers in rural and less affluent areas has also been well documented [33]. There are currently no complete national data on practicing behavioral health providers, but Ellis et al estimated the mental health workforce was about 350,000 in 2009, with psychiatrists and advanced practice psychiatric nurses in shortest supply [33]. State and federal programs designed to bring behavioral health professionals to medical underserved areas, such as the National Health Services Corps, play a vital role in connecting communities to these health care resources, but recruitment may be limited by insufficient supply. Our model estimated 6,992 additional mental health FTE were needed in health centers in 2010, which is less than 2 percent of the workforce of all providers.

A small number of vanguard health systems, including the Veterans Administration model of collaborative mental health-primary care at the White River Junction (WRJ) VA Medical Center, provide evidence to inform staffing. As of 2010, the White River Junction clinics employed one therapist, one psychiatrist, and twelve primary care physicians for every 14,000 primary care patients [26]. Compared to our model, the WRJ clinics employed significantly fewer therapists per capita, but nearly the same ratio of psychiatrists.

Our estimates are shaped and limited by health centers’ current staffing patterns. Communities will need to account for their own local preferences, partnerships, and available resources when making staffing and delivery decisions. For instance, both urban and rural communities experience a high rate of psychiatrist vacancies, which likely will hinder recruitment [34]. For these reasons, the total number of FTE needed to reach 40 million patients may be more informative than the number of FTE for each provider type.

It is also important to note primary care providers play a major role in screening, treating, and referring patients to appropriate behavioral health services. They are often the first point of contact for behavioral health needs, and they are responsible for most anti-depressants prescribed in the U.S. [35,36]. Integrating behavioral health into primary care does not reduce the need for primary care providers, but redistributes some of that care as a team of providers shares responsibility for the whole patient.

Future staffing needs, particularly as more health centers move to fully integrate behavioral health services into primary care and adopt new patient empanelment processes, are difficult to predict because staffing models and the location of behavioral health service delivery are changing. Health centers across the nation are fully integrating behavioral health into routine primary care practices and transitioning into recognized Patient-Centered Medical Homes (PCMH). Accordingly, the use of interdisciplinary primary care teams along with more robust self-management support and patient education resources will influence how care is practiced. What is clear is the need to significantly increase the behavioral health workforce, as well as evolving their competencies and skills required to effectively function in an interdisciplinary primary care team.

There are several issues that must be addressed as we work toward filling the behavioral health workforce gaps in health centers:

1) Behavioral health provider supply: The overall supply of behavioral health providers in all professional categories will likely limit health centers’ ability to recruit providers.

2) Training and education: The majority of primary care and behavioral health providers were not trained to work together. Retraining providers to adapt to the professional culture of integrated behavioral health and primary care will be necessary to maximize the effectiveness of the model.

3) Financing for sustainability: Achieving financial sustainability to continue to support integration is paramount due to the historic separation of mental health funding from physical health funding. Funding for these services will need to be protected against reallocation to other pressing needs. This requires continued federal and state investment in health centers, including the availability of health center Expanded Medical Capacity grants for behavioral health services made available under federal health center funding.

4) Measurement and evaluation: Continuing to examine the impact of integrated services, both in health centers and other sites, will shape the more widespread adoption of this new model of healthcare across the system. Assessing clinical, operational and financial outcomes upon achieving the recommended staffing is also encouraged to determine what improvements in clinical outcomes and savings are seen in the system.

Several provisions of the ACA are aimed at mitigating the shortage of behavioral health providers in community based settings, through fostering care integration or training. In addition, a joint collaboration between the federal Substance Abuse and Mental Health Services Administration and HRSA has funded community-based partnerships that promote the integration of primary and behavioral health care.

### Limitations

First, the UDS data create some limitations. The UDS includes encounters and patients for services provided off-site (i.e., not through a health center-employed clinician) but paid for by the health center. We limited our utilization analysis to grantees with behavioral health staff onsite, but we may have overestimated the number of visits a health center provider can reasonably provide if some patients from those grantees also received services offsite. Furthermore, the UDS does not break out staffing categories beyond what is provided here.

Utilization data from the UDS were significantly skewed, with a small number of grantees reporting large numbers visits per provider and/or visits per behavioral health patient. Variations in disease severity, staffing models, and treatment modalities will influence the annual number of visits per patient and per clinician. It is likely that certain health centers treat a significant number of patients with SMI, which we excluded from this study. We used median values to estimate the number of providers needed, but the model could be run with other cut-points to account for differences in service use. It is likely an underestimation of actual need but absent data on what number of visits is adequate for this population, it is at least rooted in the experience of patients receiving care in these settings. We chose the median to estimate the midpoint of those experiences across all health centers with such services. It offers a measure of equity across FQHCs even if it doesn’t achieve adequacy. That is, it offers a measure of what workforce would be needed to achieve the same level of service across all health centers given the evidence-based prevalence of need for such care. This analysis also used only one year of data, though it was the most recent at the time. Changing staffing models may be apparent when exploring future or more years of UDS.

Second, we acknowledge that under broad insurance expansion, most uninsured patients will likely gain coverage through Medicaid, though many will also gain private insurance through the exchanges. Because our two categories of insurance represent applied benchmarks for assessing workforce needs, we recognize that our estimates under these insurance-based assumptions are relatively conservative.

Third, our estimates are still based on current staffing patterns in FQHC. Research has highlighted that health centers often experience staffing shortages and that they serve more patients per provider compared to other providers [37]. It remains unclear whether the allocation of behavioral health positions would be made according to historic staffing patters or a new model. Further, estimates for staffing in general may be low given the presumption that by expanding behavioral health services, more patients may be retained over time in the FQHC.

Fourth, data from the NSDUH may likely underestimate need for behavioral health in FQHCs as it is based on general estimates from a national survey not necessarily reflective of the population seen in FQHCs.

Finally, because our study data sources were constrained to those age 12 and older, we likely underestimate behavioral health service needs for the full range of patients served in these settings and neglect specific counts of child behavioral health specialists.

## Conclusions

Integrating behavioral health providers into primary care creates a promising opportunity to better meet the behavioral health needs of health center patients by reducing the fragmentation that creates so many barriers for vulnerable populations. More mental health is seen in primary care than in any other setting, and the number of primary care patients served by community health centers is expected to expand significantly. As health centers grow, so will access to needed health care services, but only with appropriate planning. We need a better understanding of how many behavioral health providers are needed in health centers. This paper adds to the literature by proposing one way to assess the behavioral workforce need as well as the implications of these recommendations for health policy.

## Competing interests

This publication was supported by Grant/Cooperative Agreement Number U30CS16089 from the Health Resources and Services Administration, Bureau of Primary Health Care (HRSA/BPHC). Its contents are solely the responsibility of the authors and do not necessarily represent the official views of HRSA/BPHC.

## Authors’ contributions

BB, BM, MP, RP, and AB developed the research question and research design. BB and BM worked with SP to conduct the analyses and produce the first draft of the manuscript. MP, AB, EG and RP reviewed the initial draft and contributed to subsequent drafts. All authors read and approved the final manuscript.

## Pre-publication history

The pre-publication history for this paper can be accessed here:

http://www.biomedcentral.com/1472-6963/13/245/prepub
